# Reliability and development of a new classification of lumbosacral spondylolisthesis

**DOI:** 10.1186/1748-7161-3-19

**Published:** 2008-12-10

**Authors:** Jean-Marc Mac-Thiong, Hubert Labelle, Stefan Parent, Michael Timothy Hresko, Vedat Deviren, Mark Weidenbaum, members of the Spinal Deformity Study Group

**Affiliations:** 1 Department of Surgery, University of Montreal, Montreal, Canada; 2 Division of Orthopedic Surgery, Hôpital du Sacré-Coeur, Montreal, Canada; 3 Division of Orthopedic Surgery, CHU Sainte-Justine, Montreal, Canada; 4 Department of Orthopaedic Surgery, Boston Children Hospital, Harvard University, USA; 5 Department of Orthopaedic Surgery, San Francisco General Hospital, UCSF, SF, USA; 6 Department of Orthopaedic Surgery, Columbia University, New York Presbyterian Hospital, NY, USA

## Abstract

**Background:**

A classification of lumbosacral spondylolisthesis has been proposed recently. This classification describes eight distinct types of spondylolisthesis based on the slip grade, the degree of dysplasia, and the sagittal sacro-pelvic balance. The objectives of this study are to assess the reliability of this classification and to propose a new and refined classification.

**Methods:**

Standing posteroanterior and lateral radiographs of the spine and pelvis of 40 subjects (22 low-grade, 18 high-grade) with lumbosacral spondylolisthesis were reviewed twice by six spine surgeons. Each radiograph was classified based on the slip grade, the degree of dysplasia, and the sagittal sacro-pelvic balance. No measurements from the radiographs were allowed. Intra- and inter-observer reliability was assessed using kappa coefficients. A refined classification is proposed based on the reliability study.

**Results:**

All eight types of spondylolisthesis described in the original classification were identified. Overall intra- and inter-observer agreement was respectively 76.7% (kappa: 0.72) and 57.0% (kappa: 0.49). The specific intra-observer agreement was 97.1% (kappa: 0.94), 85.0% (kappa: 0.69) and 88.8% (kappa: 0.85) with respect to the slip grade, the degree of dysplasia, and the sacro-pelvic balance, respectively. The specific inter-observer agreement was 95.2% (kappa: 0.90), 72.2% (kappa: 0.43) and 77.2% (kappa: 0.69) with respect to the slip grade, the degree of dysplasia, and the sacro-pelvic balance, respectively.

**Conclusion:**

This study confirmed that surgeons can classify radiographic findings into all eight types of spondylolisthesis. The intra-observer reliability was substantial, while the inter-observer reliability was moderate mainly due to the difficulty in distinguishing between low- and high-dysplasia. A refined classification excluding the assessment of dysplasia, while incorporating the assessment of the slip grade, sacro-pelvic balance and global spino-pelvic balance is proposed, and now includes five types of lumbosacral spondylolisthesis.

## Background

**Figure 1:**
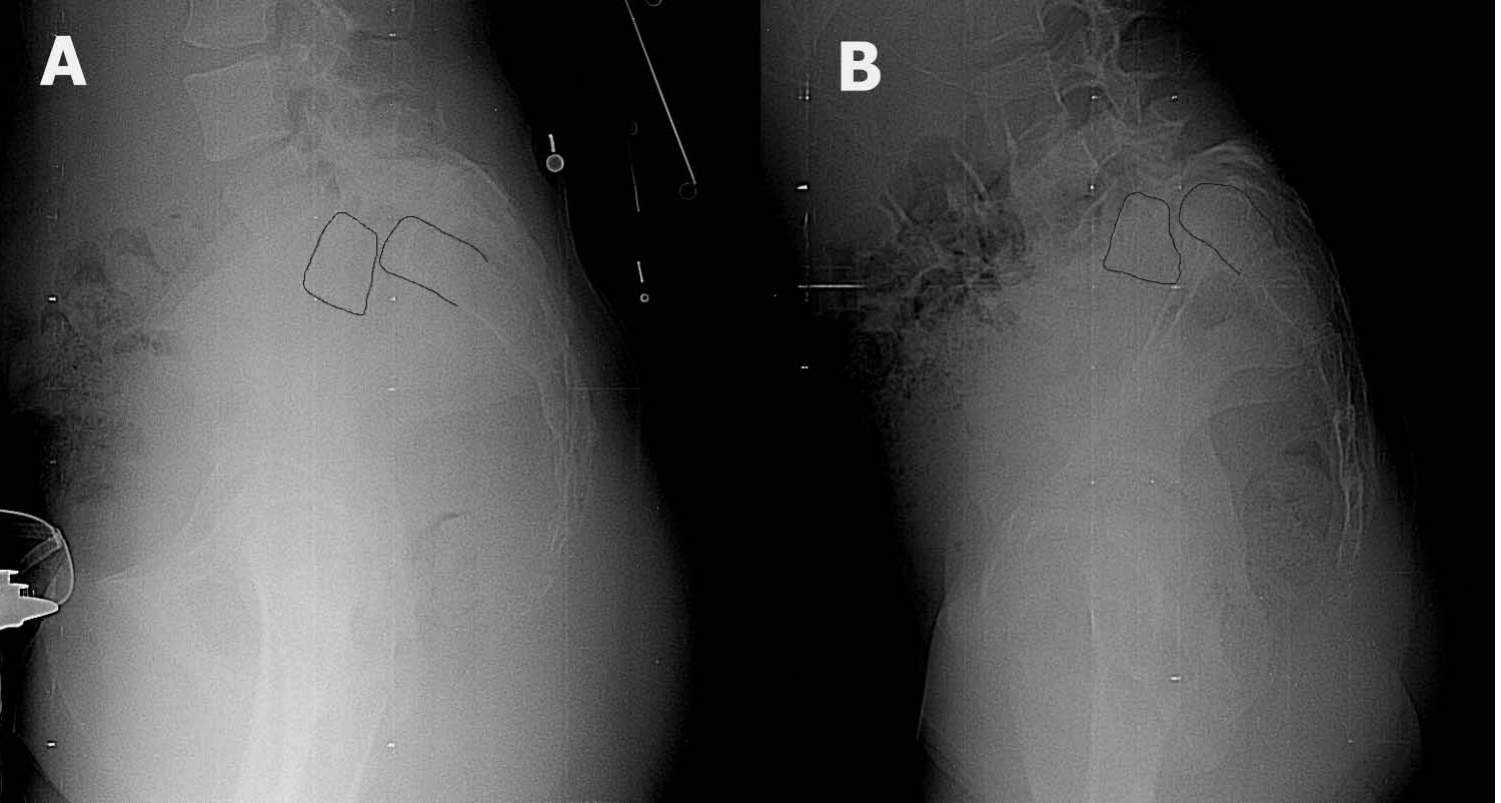
Two patients with high-grade spondylolisthesis. The first patient (A) has relatively rectangular L5 and S1 vertebrae. The lumbosacral kyphosis is not significant and the sacral slope is high. In the second patient (B), the L5 vertebra is trapezoidal and there is significant doming of sacrum. Lumbosacral kyphosis is important and the sacrum is more vertical.

Spondylolisthesis has been commonly described using the classification system developed by Wiltse et al. [1] which divides spondylolisthesis into five types. Type I (dysplastic) is associated with congenital dysplasia of L5 or the sacrum, while type II (isthmic) spondylolisthesis involves a defect in the pars interarticularis. Marchetti and Bartolozzi [2] developed a classification system which distinguished developmental from acquired spondylolisthesis and which further divided developmental spondylolisthesis into low- and high-dysplastic. Neither of these two classification systems [1,2] were specifically designed for treatment planning of spondylolisthesis. In addition, slip grade is only one of many abnormalities involved in spondylolisthesis (Figure 1). Despite this, surgical guidelines and outcome studies for spondylolisthesis are primarily based on slip grade [3-6]. Furthermore, these classifications [1,2] do no take sagittal sacro-pelvic balance into account, although many more recent studies have suggested its importance in the evaluation, progression and treatment of spondylolisthesis [7-20]. These findings may in part explain the wide variability reported in the literature concerning surgical techniques and outcomes for spondylolisthesis, and support our opinion that an optimal algorithm for treatment has yet to be defined.

Recently, Mac-Thiong and Labelle [21] proposed a new classification of spondylolisthesis specifically intended to guide the evaluation and treatment of lumbosacral spondylolisthesis. This classification clarifies the concept of low- and high-dysplasia introduced by Marchetti and Bartolozzi [2] and incorporates recent knowledge in the study of sagittal sacro-pelvic balance and morphology. Eight distinct types of spondylolisthesis are described in this classification based on the following: 1) slip grade (low- vs. high-grade), 2) degree of dysplasia (low- vs. high-dysplastic), and 3) sagittal sacro-pelvic balance. The classification organizes groups and subgroups in an ascending order of severity to develop an associated intuitive surgical algorithm of increasingly complex surgery as the severity of the spondylolisthesis increases.

**Figure 2:**
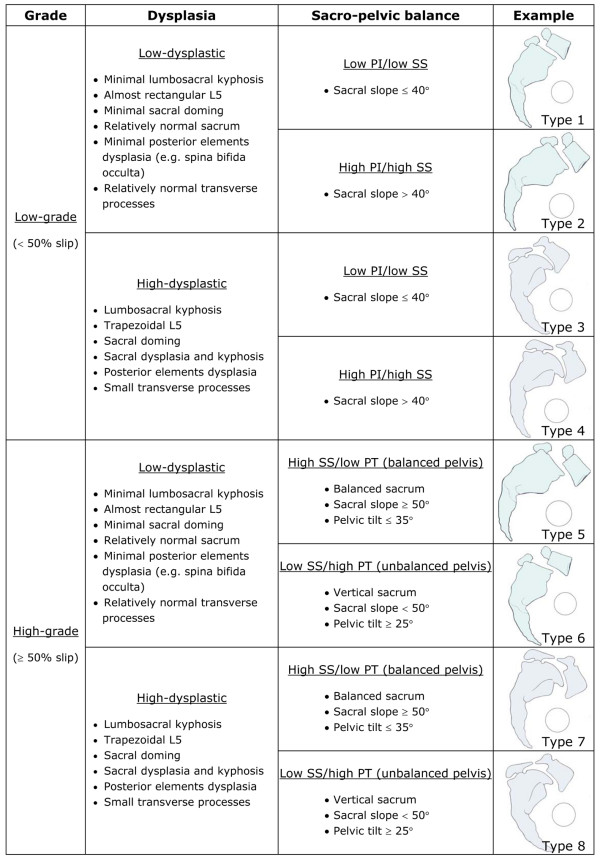
Original classification system of lumbosacral spondylolisthesis.

Using a simplified version of the original classification (Figure 2), good intra- and inter-observer agreement of respectively 90% and 75% were found in a preliminary report [22]. However, since the observers involved in that study were the two initial developers of the classification system, further verification with additional observers is needed to validate the proposed simplified classification. The main objective of this study is therefore to assess the reliability of the classification. Based on the results from the reliability study and on recent findings from the Spinal Deformity Study Group (SDSG), a new and refined SDSG classification of spondylolisthesis will also be proposed.

## Methods

The radiological files of all patients with lumbosacral developmental spondylolisthesis seen for the first time at the spine clinic of a paediatric hospital between July 1^st ^2001 and March 1^st ^2006 were reviewed. Patients were defined as potential subjects for inclusion in the study if they had postero-anterior (PA) and lateral (LAT) standing radiographs of the spine and pelvis showing both femoral heads. All subjects with a history or clinical signs of hip, pelvic or lower limb disorder were excluded. For patients who had undergone surgical treatment of spondylolisthesis, only the preoperative radiographs were considered. All 18 patients with high-grade spondylolisthesis were included. Twenty-two patients were randomly selected from the remaining 108 patients with low-grade spondylolisthesis. Thus a total of 40 subjects with spondylolisthesis (18 high-grade and 22 low-grade) were available for this study. All radiographs used in the current study were retrieved from the PACS system by an independent observer and there was no header or any information on the radiographs in order to minimize potential sources of bias. The mean percentage of slip was 16 ± 8% (range: 4–44%) for subjects with low-grade spondylolisthesis and 80 ± 17% (range: 53–100%) for subjects with high-grade slips. The mean age was 14.7 ± 2.9 years (range: 7.9–20.0 years).

Six spine surgeons from four different institutions classified all 40 subjects twice (at a one week interval), based on digital standing PA and LAT radiographs of the spine and pelvis viewed on a computer screen. The observers were allowed to view the radiographs with the software of their choice. They classified the spondylolisthesis for each subject into one of eight types described by the classification system provided in Figure 2. In this classification system, the slip grade, the degree of dysplasia and the sacro-pelvic balance are determined visually. Quantitative criteria for slip grade and sacro-pelvic balance are given as a reference, while qualitative criteria are provided for the degree of dysplasia. However, observers were not allowed to make any measurements on the radiographs but only made visual estimates in order to classify each case.

Statistical analysis was performed by a biostatistician from PhDx Systems Inc (Albuquerque, NM). Classification reliability was assessed by calculating the intra- and inter-observer percentage of agreement, as well as the kappa coefficients. The resulting kappa values were interpreted based on the recommendations of Landis and Koch [23] (< 0: poor agreement; 0–0.20: slight agreement; 0.21–0.40: fair agreement; 0.41–0.60: moderate agreement; 0.61–0.80: substantial agreement; 0.81–1: almost perfect agreement).

## Results

Four of six observers identified all eight types of spondylolisthesis described in the classification (Table 1). Two observers did not identify any radiograph with a type 3 spondylolisthesis (Table 1). The results from the reliability study are presented in Table 2. The overall intra-observer percentage of agreement was 77%, with an associated kappa value of 0.72 corresponding to substantial agreement. The overall inter-observer percentage of agreement and kappa value were respectively 57% and 0.49, indicating moderate agreement. Of all 40 radiographs, 10 (nine low-grade and one high-grade) resulted in perfect agreement between all six observers, and eight (two low-grade and six high-grade) resulted in complete agreement among five observers. In total, 12 (55%) low-grade and seven (39%) high-grade subjects showed agreement between at least five observers. Ten radiographs had agreement between four observers, while the remaining 12 resulted in agreement between two or three observers only.

**Table 1:** Number of cases identified by each observer for each spondylolisthesis type

	Spondylolisthesis type							
	
	Type 1	Type 2	Type 3	Type 4	Type 5	Type 6	Type 7	Type 8
Observer 1	14	7	0	2	1	4	2	10
Observer 2	6	6	3	7	3	5	2	8
Observer 3	6	8	2	5	5	6	2	6
Observer 4	7	9	1	6	1	1	3	12
Observer 5	9	8	4	4	1	2	6	6
Observer 6	8	8	0	5	4	6	2	7

**Table 2:** Intra- and inter-observer reliability

	Intra-observer reliability		Inter-observer reliability	
		
	% agreement	Kappa	% agreement	Kappa
Spondylolisthesis type	76.7%	0.72	57.0%	0.49
Slip grade	97.1%	0.94	95.2%	0.90
Degree of dysplasia	85.0%	0.69	72.2%	0.43
Sacro-pelvic balance	88.8%	0.85	77.2%	0.69

**Figure 3:**
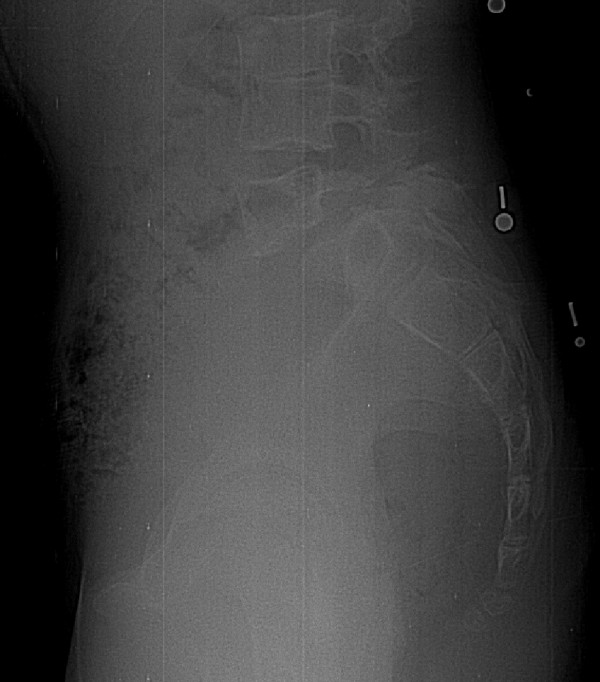
A case associated with disagreement between observers regarding the slip grade. Only the lumbosacral region from the original radiographs is provided.

Specific intra- and inter-observer reliability for the determination of grade, degree of dysplasia and sacro-pelvic balance are also provided in Table 2. Reliability was highest with respect to slip grade, for which agreement was almost perfect with intra- and inter-observer kappa values of 0.94 and 0.90, respectively. Disagreement between observers concerning the slip grade occurred for only four cases. Figure 3 shows an example of a case with a 44% slip. However, disagreement regarding the slip grade occurred, as four observers classified the case as a low-grade slip, while two observers classified it as a high-grade slip.

**Figure 4:**
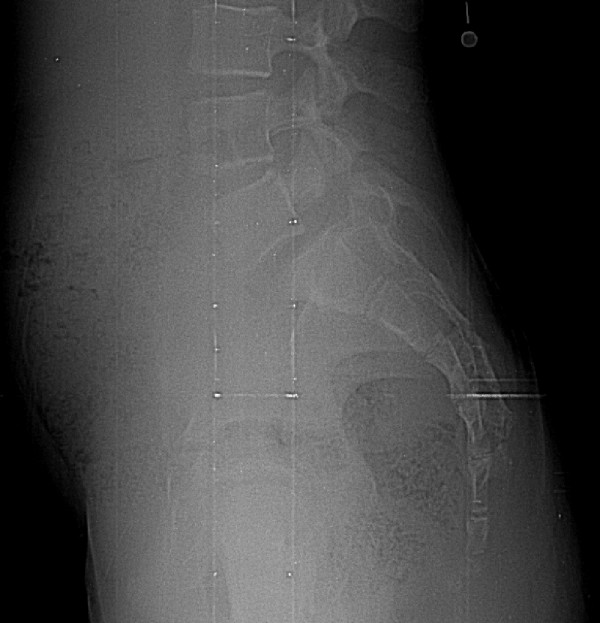
A case associated with disagreement between observers regarding the sacro-pelvic balance for this low-grade spondylolisthesis. Only the lumbosacral region from the original radiographs is provided.

With regard to assessment of sacro-pelvic balance, there was almost perfect intra-observer agreement (kappa: 0.85) as well as substantial inter-observer agreement (kappa: 0.69). In the 36 cases wherein all observers agreed on grade, seventeen had agreement between all six observers, and fourteen had agreement between five observers. The remaining five (four low-grade and one high-grade) cases showed agreement for only four observers. Figure 4 shows an example of a case with disagreement regarding the sacro-pelvic balance. All observers agreed that the spondylolisthesis was low-grade. However, four observers classified the case as a high pelvic incidence/high sacral slope (high PI/high SS) type of sacro-pelvic balance, while two observers classified it as a low pelvic incidence/low sacral slope (low PI/low SS) type. Post-hoc direct measurement on the radiograph revealed a borderline sacral slope of 43 degrees.

The lowest reliability was associated with the degree of dysplasia (low- vs. high-dysplastic) wherein intra-observer agreement was substantial (kappa: 0.69) but inter-observer agreement was only moderate (kappa: 0.43). Seventeen cases resulted in agreement between all six observers, and eight cases had agreement for five observers. However, 15 subjects resulted in agreement for only three or four of the six observers, indicating that in these cases agreement was only related to chance. Of those 15 subjects, nine had low-grade spondylolisthesis and six had high-grade spondylolisthesis.

**Figure 5:**
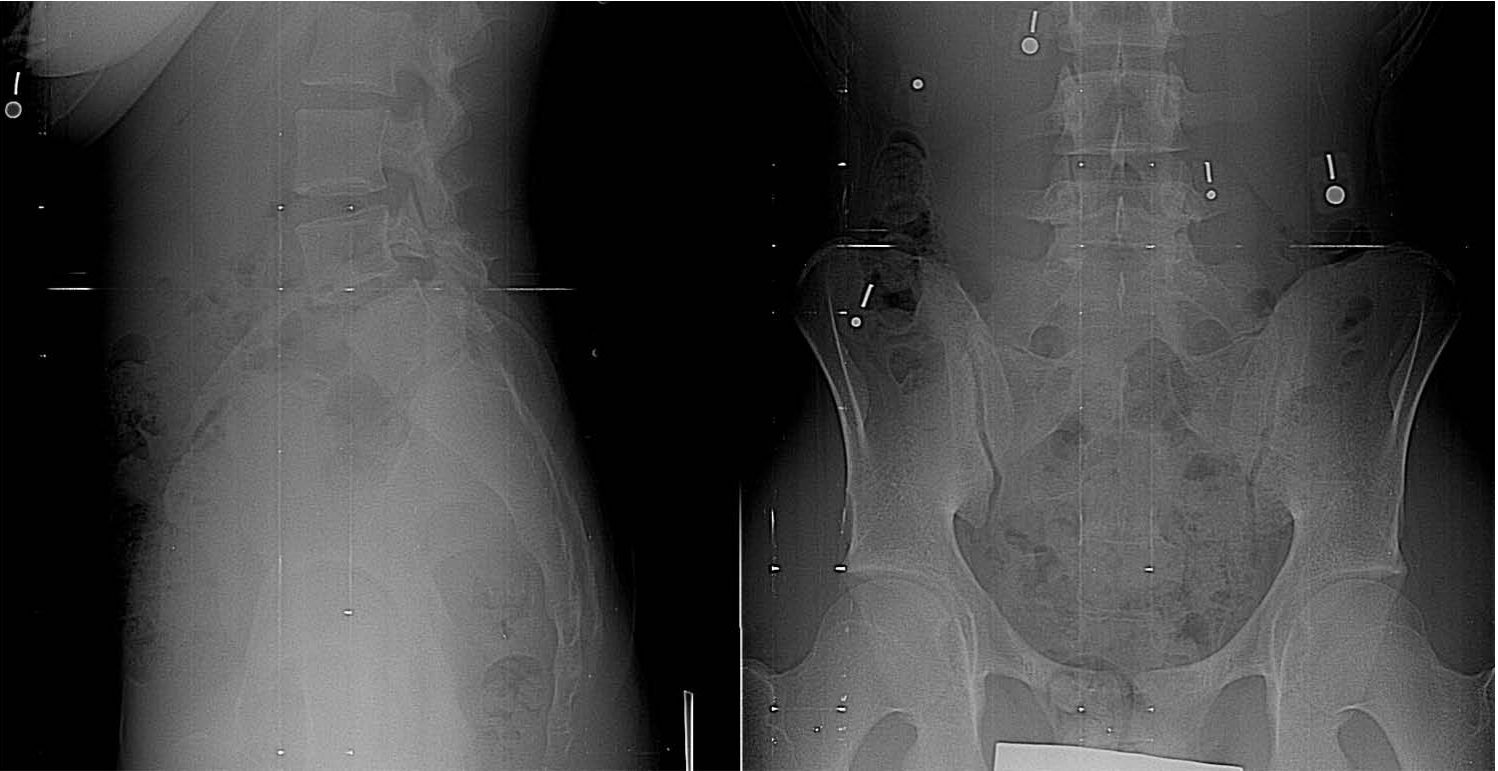
A case of low-grade spondylolisthesis for which there was complete intra- and inter-observer agreement among all observers (type I spondylolisthesis). Only the lumbosacral region from the original radiographs is provided.

**Figure 6:**
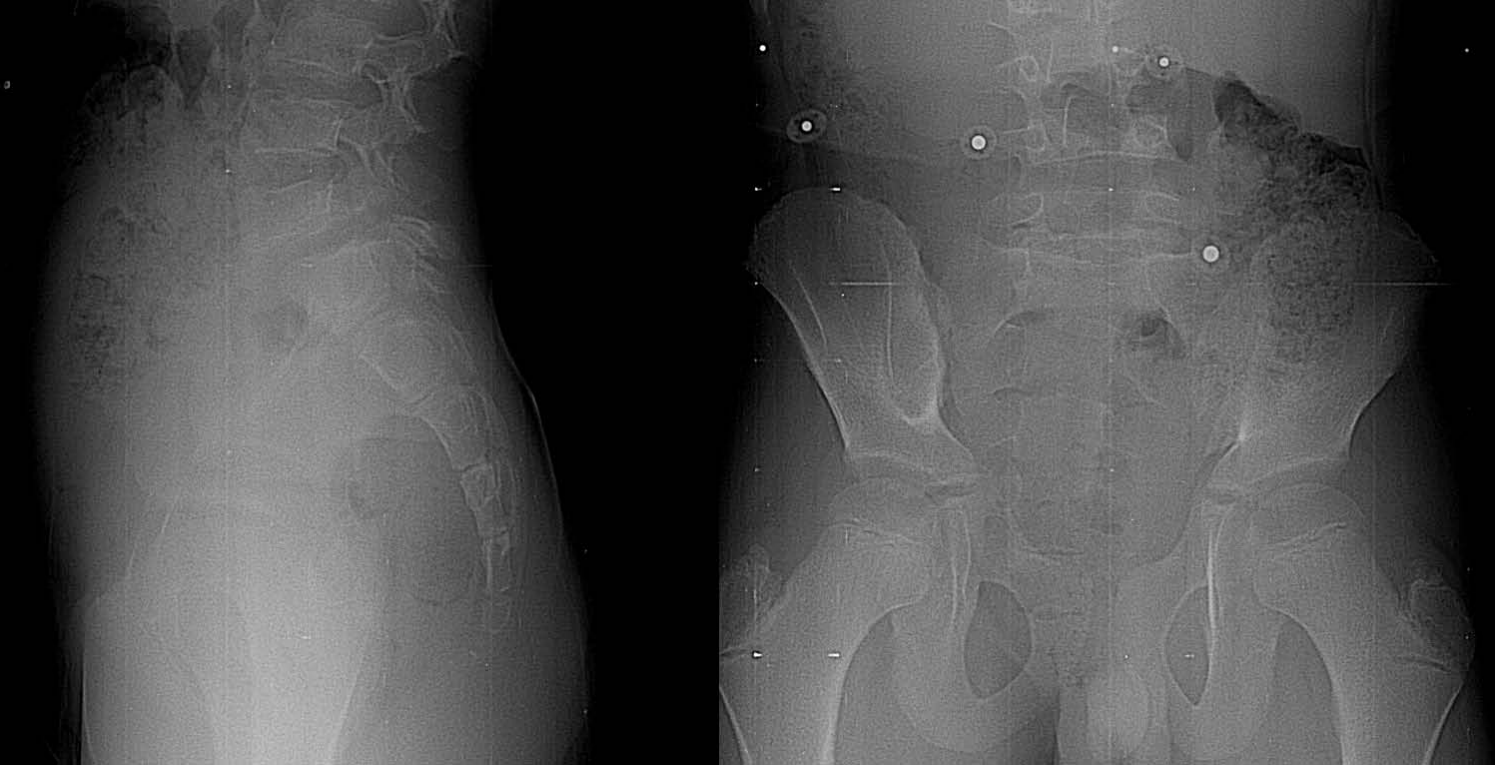
A case of low-grade spondylolisthesis for which there was intra-observer agreement in five of six observers with respect to the degree of dysplasia. However, the inter-observer agreement in the determination of the degree of dysplasia was poor because three observers classified the case as a type I (low-dysplastic), while the three other observers classified it as a type III (high-dysplastic) spondylolisthesis. Only the lumbosacral region from the original radiographs is provided.

Figure 5 shows an example of a case with complete intra- and inter-observer agreement. All observers classified the case as a Type I spondylolisthesis. In contrast, Figure 6 demonstrates a case where there was intra-observer agreement in five of six observers, but where inter-observer agreement was poor. All observers agreed that the spondylolisthesis was a low-grade and low PI/low SS type, but three observers classified the case as a Type I, while the three other observers classified it as a Type III spondylolisthesis. This disagreement occurred due to lack of agreement in determination of the degree of dysplasia.

## Discussion

This study evaluated the reliability of a classification previously proposed for lumbosacral spondylolisthesis [21,22]. First, it confirmed that surgeons can classify radiographic findings into all eight types in a random sample of subjects with spondylolisthesis. Second, the classification system yielded substantial intra-observer reliability (kappa: 0.72) and a moderate inter-observer reliability (kappa: 0.49). These values are similar to those reported in comparing the reliability of the King et al. [24] and Lenke et al. [25] classification systems for adolescent idiopathic scoliosis [26], although the current classification is a "visual semi-quantitative" classification system. Richards et al. [26] found intra- and inter-observer kappa coefficients of 0.81 and 0.61 respectively for the King et al. classification, while the intra- and inter-observer kappa values were respectively 0.60 and 0.50 for the Lenke et al. classification. Other studies have also reported kappa values in the same range for the King et al. classification (intra-observer kappa: 0.64; inter-observer kappa: 0.44) [27] and the Lenke et al. classification (intra-observer kappa: 0.73; inter-observer kappa: 0.62) [28].

It is expected that the clinical reliability of the classification could be improved if direct measurements on the radiographs were made. Indeed, the fact that no measurements were allowed in this study may explain most of the cases for which there was disagreement concerning slip grades around 50% (Figure 3), although the intra- and inter-observer reliability was almost perfect for slip grade assessment. Measurement of pelvic tilt and sacral slope could undoubtedly improve the reliability associated with sagittal sacro-pelvic balance, as the associated intra-observer reliability was almost perfect but the inter-observer reliability was substantial. Consequently, future development of the classification should attempt to increase the reliability concerning the assessment of the slip grade and of the sacro-pelvic balance, while keeping the classification simple to use clinically. In the future, classification of cases will be made using a software that requires identification of only a limited number of anatomical landmarks and that automatically provides the slip grade and sacro-pelvic balance.

Furthermore, this study demonstrated that most of the disagreement in the classification centered in determination of the degree of dysplasia when using only qualitative criteria. Although intra-observer agreement for the degree of dysplasia was substantial (kappa: 0.69), inter-observer agreement was only moderate (kappa: 0.43). As seen in Figure 6, each observer developed his own interpretation to discriminate low- from high-dysplasia. The problem with determination of the degree of dysplasia is therefore related to the high variability between observers concerning the interpretation of the criteria. This source of inter-observer disagreement could surely be reduced by the implementation of quantitative criteria such as those proposed in the original classification [21], although these could be difficult to apply clinically in their present form due to their complexity. It is possible that determination of the degree of dysplasia with the aid of CT scan images and/or MRI could have improved the reliability of the classification.

The reliability for classifying patients could also be improved by using Ferguson and lateral lumbosacral junction views, in addition to the PA and LAT radiographs of the complete spine and pelvis that were used in this study. The reduced visibility of the lumbosacral junction on PA and LAT radiographs of the complete spine and pelvis might explain part of the discrepancies in this study, especially for high-grade subjects.

### Proposal of the refined SDSG classification of lumbosacral spondylolisthesis (Table 3)

**Table 3:** SDSG classification system of lumbosacral spondylolisthesis

**Slip grade**	**Sacro-pelvic balance**	**Spino-pelvic balance**	**Spondylolisthesis type**
Low-grade	Normal pelvic incidence	--	Type 1
	
	High pelvic incidence	--	Type 2

High-grade	Balanced	--	Type 3
	
	Unbalanced	Balanced	Type 4
		
		Unbalanced	Type 5

In the perspective of developing a surgical treatment algorithm for lumbosacral spondylolisthesis, it is important to propose a classification system that is simple to use clinically and highly reliable. Due to the moderate inter-observer reliability for the assessment of the degree of dysplasia and also due to the difficulty to define reliable quantitative criteria for dysplasia, we have decided to exclude the assessment of dysplasia from the classification system.

In addition, recent work from the SDSG has led us to modify the determination of spino-pelvic balance in low-grade spondylolisthesis. A study (unpublished data, manuscript under preparation) conducted on 257 patients with low-grade spondylolisthesis and using K-means cluster analysis of sacro-pelvic parameters (pelvic incidence, sacral slope and pelvic tilt) showed that these patients were divided into two distinct groups: a group with normal or near normal pelvic incidence (< 60°) and a group with high pelvic incidence (≥ 60°). The low PI/low SS and high PI/high SS groups mentioned in the original classification system are in fact subtypes of the two groups recently described (pelvic incidence < 60° vs. pelvic incidence ≥ 60°).

Also, it is now recognized that preservation or restoration of an adequate global sagittal balance is of prime importance in the management of spinal deformity, so that assessment of global balance has been introduced into the SDSG classification. Two studies [29,30] have demonstrated that sagittal balance is related to the health-related quality of life in spinal deformity. Furthermore, from their study on 120 controls, 91 subjects with low-grade spondylolisthesis and 40 subjects with high-grade spondylolisthesis, Mac-Thiong et al. [31] have shown the importance of evaluating the global sagittal balance with respect to the position of the femoral heads (spino-pelvic balance).

The revised classification of lumbosacral spondylolisthesis supported by the SDSG is based on three important characteristics that can be assessesd from the preoperative imaging studies: 1) the grade of slip, 2) the sacro-pelvic balance, and 3) the global spino-pelvic balance. Accordingly, five different types of spondylolisthesis have been identified (Table 3). To classify a patient, the degree of slip is quantified first from the lateral radiograph, in order to determine if it is low-grade (grades 0, 1 and 2, or < 50% slip) or high-grade (grades 3, 4 and spondyloptosis, or ≥ 50% slip). Next, the sagittal balance is measured by determining sacro-pelvic and global spino-pelvic balance, using measurements of pelvic incidence (PI), sacral slope (SS), pelvic tilt (PT) and the C7 plumbline.

**Figure 7:**
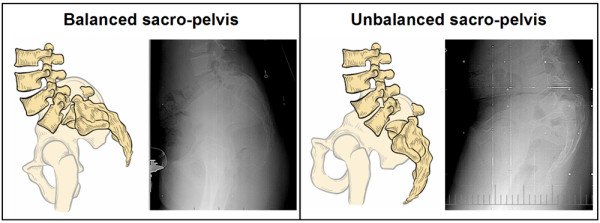
**Two types of sacro-pelvic balance in high-grade spondylolisthesis**. (modified from Hresko et al., Spine 2007)

**Figure 8:**
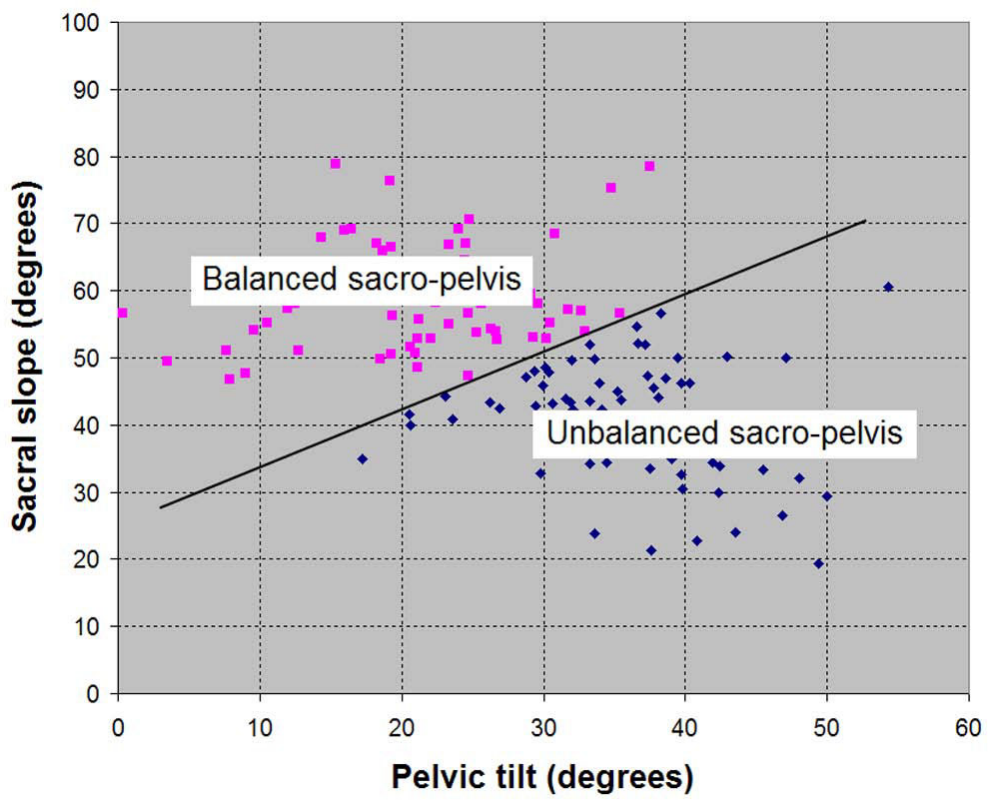
Determination of sacro-pelvic balance in high-grade spondylolisthesis (modified from Hresko et al., Spine 2007).

In high-grade spondylolisthesis, sacro-pelvic balance is assessed based on the findings of Hresko et al [10] (Figure 7). Each subject is classified as high SS/low PT (balanced sacro-pelvis) or low SS/high PT (unbalanced sacro-pelvis) according to Figure 8, which illustrates the relationship between SS and PT in high-grade spondylolisthesis. When SS and PT are located above the threshold line, the subject is classified as high SS/low PT. On the other hand, when SS and PT are located below the threshold line, the subject is classified as low SS/high PT. For low-grade spondylolisthesis, two types of sacro-pelvic balance can be found: a subgroup with normal or near normal PI values (< 60°) and a subgroup with high PI (≥ 60°). Finally, global spino-pelvic balance is determined using the C7 plumbline. If this line falls over or behind the femoral heads, the spine is balanced, while if it lies in front of both femoral heads, the spine is unbalanced. In our experience, the spine is almost always balanced in low-grade and in high-grade spondylolisthesis with a balanced sacro-pelvis and therefore, spinal balance needs to be measured mainly in high-grade deformities with an unbalanced pelvis.

## Conclusion

This study evaluated the reliability of a classification previously proposed for lumbosacral spondylolisthesis. The reliability study showed suboptimal inter-observer reliability regarding the assessment of dysplasia, but excellent reliability for the slip grade and sacro-pelvic balance. A refined classification excluding the assessment of dysplasia, while incorporating the assessment of the slip grade, sacro-pelvic balance and global spino-pelvic balance is proposed, and now includes five types of lumbosacral spondylolisthesis. In addition, the reliability of the classification is expected to increase with direct measurements from the radiographs.

## Competing interests

This research was assisted by support from the Spinal Deformity Study Group. This research was funded by an educational/research grant from Medtronic Sofamor Danek.

## Authors' contributions

JMMT and HL were responsible for the design of the study, as well as the data analysis. All authors participated in the classification of all cases, as well as in the preparation and approval of the final manuscript.
